# Effect of ΔNp63β on cell cycle and apoptosis in T98G cells

**DOI:** 10.55730/1300-0144.5919

**Published:** 2024-06-20

**Authors:** Buse TÜREGÜN ATASOY, Fikret ŞAHİN

**Affiliations:** Department of Microbiology, Faculty of Medicine, Ankara University, Ankara, Turkiye

**Keywords:** p63, T98G, cell signaling, apoptosis

## Abstract

**Background/aim:**

The p53 protein, a crucial tumor suppressor, governs cell cycle regulation and apoptosis. Similarly, p63, a member of the p53 family, exhibits traits of both tumor suppression and oncogenic behavior through its isoforms. However, the functional impact of ΔNp63β, an isoform of the p63 protein, on human glioma cancer cells like T98G cells remains poorly understood, representing the novelty of this study in the current literature.

**Materials and methods:**

Employing the pRetroX-Tet-On vector system, the apoptotic effects of ΔNp63β on T98G cell lines was investigated and its influence on the cell cycle was assessed. Initially, an rtTA-expressing vector, a component of the pRetroX-Tet-On system, was established in the T98G cell lines. Subsequently, the ΔNp63β cDNA was cloned into the Retropur Tight retroviral vector and transfected into T98G cells containing the pRetroX-Tet-On system for functional analysis. The gene expression and cell cycle regulation were evaluated through reverse-transcription polymerase chain reaction and flow cytometry, determining protein translation via western blotting. 3-(4,5-dimethylthiazol-2-yl)-2,5-diphenyltetrazolium bromide assay and β-galactosidase cell staining were employed to assess the cytotoxicity and senescence of ΔNp63β, respectively

**Results:**

The overexpression of ΔNp63β in the T98G cells correlated with increased cell viability and altered cell cycle regulation, notably upregulating the p21 expression independent of p53. Caspase-3/7 activity analyses showed no changes in the apoptotic genes but revealed an increase in antiapoptotic gene expression. Surprisingly, cell death in the ΔNp63β-overexpressing T98G cells did not occur through apoptosis as anticipated. Instead, it resulted from the cytotoxic effects of the ΔNp63β protein.

**Conclusion:**

Δp63β increased the p21 levels, induced cell death, and caused cell cycle arrest at the G1 phase, while exhibiting antiapoptotic properties and promoting senescence. Unexpectedly, overexpression of Δp63β in T98G cells led to significant cell death, potentially through necrosis rather than apoptosis, suggesting a complex role for Δp63β in cell cycle regulation and tumor suppression.

## 1. Introduction

P63, a protein discovered in 1998, shares structural similarities with p53, the renowned tumor suppressor gene. Proteins encoded by the p63 gene exhibit domains within their protein structure similar to those found in p53, including the N-terminal transactivation, DNA binding, and carboxy-oligomerization domains. However, distinct isoforms with entirely different functions are generated through alternate promoters or alternative splicing beyond these commonly shared domains [1–3]. p63, a member of the p53 family, is recognized for its distinct characteristics compared to p53 isoforms, which are acknowledged for their tumor suppressor functions. Notably, while p53 frequently undergoes mutations that lead to losing its tumor suppressor abilities in various cancers, p63 exhibits a different mutation rate and distinct functionalities [4]. P63 forms two isoforms with and without a transactivation domain and then five different isoforms by alternative splicing at the carboxyl terminus [5–7]. TAp63 functions as a tumor suppressor, promoting cell death and cell cycle arrest, while ΔNp63 is predominantly recognized as oncogenic due to its tendency to be overexpressed in cancer. These distinct characteristics categorize these two isoforms as exerting opposing effects [8–11]. Many studies have shown that the role of p63 is more directly related to tumor development and differentiation than the tumor suppressor p53 [12]. Moreover, the p63 protein functions as a transcription factor due to its sequence-specific DNA-binding properties [2,5,13]. TAp63 isoforms activate transcription through p53 DNA binding sites and heterologous promoters, as well as the transcription of target genes with its promoter and endogenous promoters of the p53 target gene, like p21. Alpha isoforms of p63 are also among the activators of p53 target genes involved in cell cycle arrest and apoptosis [14–17]. Moreover, p63 was reported to be responsible for the transactivation of genes responsible for apoptosis [9]. The observation that p63 is predominantly unaltered in mutation analyses among humans, coupled with its overexpression in specific cancer types, has sparked debates surrounding its classification as a tumor suppressor akin to p53. The discussions regarding its role as either a tumor suppressor or an oncogene have underscored the dichotomous functions exhibited by its distinct isoforms [9,18]. It has been shown that ΔNp63 inhibits p53, while TAp63 induces apoptosis through the transactivation of p53 [9,19,20]. p63 plays a critical role in the development of normal epithelial cells. It has also been shown to play an essential role in the pathogenesis of cancers originating from epithelial cells, especially epithelial cell-derived skin, lung, breast and head and neck cancers [34]. In clinically documented gliomas displaying epithelial differentiation, ΔNp63 has demonstrated efficacy within glioma tumors [35]. Glioblastoma multiforme (GBM), among the most lethal forms of brain tumors, accounts for half of the fatalities associated with malignant brain neoplasms. Despite surgical intervention and chemoradiotherapy, survival rates remain relatively brief. Regulation of p63 expression offers hope for the possibility of treatment [32]. Numerous investigations have demonstrated the multifaceted regulation of p63 expression by various signaling pathways. Notably, studies have elucidated that the inhibition of epidermal growth factor receptor (EGFR) signaling, mediated through the phosphoinositide 3-kinase (PI3K)/protein kinase B (AKT)/mammalian target of the rapamycin (mTOR) pathway, leads to a diminished expression of ΔNp63α [31]. Inhibition of the TP63/TRAF1/BIRC3/Bcl-2 signaling cascade through the p63 pathway is conjectured to modulate the proliferation, metastasis, and invasion of glioma cells [32]. Furthermore, the association between the p63 protein and signal transducer and activator of transcription 3 (STAT3) is emphasized, positing significant roles in preserving cancer stem cells and the carcinogenesis of diverse cellular lineages [33]. The multifaceted involvement of p63 in crucial cellular processes, such as cell proliferation, DNA damage control, cell cycle regulation, apoptosis, ageing, and the onset of cancer metastasis, highlights the significance of studying p63. Despite extensive research on the α isoforms of p63, which encompass numerous variants, detailed data on the ΔNp63β isoform remains limited. Based on this information, a paucity of literature explicitly addresses the ΔNp63β isoform of p63, presumed to exert efficacy in T98G cells exhibiting GBM with epithelial differentiation. In the current study, the impacts of the ΔNp63β isoform on gliomas were investigated. Consequently, this study aimed to explore the gene and protein expressions of potential target genes, especially those associated with the p53 protein and investigate the phenotypic effects induced by the ΔNp63β isoform in T98G glioma cells.

## 2. Materials and methods

### 2.1. Isolation and cloning p63 isoform

The ΔNp63β isoform was isolated from epidermoid carcinoma cell HTB-33 (ME-180 ATCC HTB33) using a Phusion High-Fidelity PCR Kit (Thermo Fisher Scientific Inc., Waltham, MA, USA). The isolated isoform was initially inserted into the pBluescriptSK(+) (pBSK) cloning vector and validated through sequence analysis employing M13F and M13R primers. The isoform confirmed by sequence analysis was then inserted into a tetracycline-induced-overexpressing retroviral vector system (pRetroX-Tight-Pur; Clontech Laboratories Inc., Mountain View, CA, USA).

### 2.2. Preparation of the cell lines

T98G cells (ATCC CRL-1690), a fibroblast-like cell isolated from the brain of a GBM, were used. The cells were incubated in Dulbecco’s minimal essential medium (DMEM) (Gibco Scientific, Grand Island, USA), 10% fetal calf serum (Hyclone Laboratories LLC, Logan, UT, USA), and 1% antibiotic (penicillin-streptomycin) (Gibco Scientific) and 1% L-glutamine (Invitrogen Life Technologies, Waltham, MA, USA) at 37 °C 5% CO_2_. The cell DMEMs were changed to tetracycline (100 ng/mL) DMEM every 24 h for 2 days.

### 2.3. Cell transfection and Luciferase reporter assay

The pRetroX-Tet-On vector (Clontech Laboratories Inc.) expressing rtTA was transfected together with retroviral packaging plasmids (pCMV-VSV-G; Addgene; plasmid 8454 and PUMVC; Addgene; plasmid 8449) into the Hek293-T (ATCC CRL-3216) cell line, one of the cells with the best particle formation of the virus, using a Calcium-Phosphate Transfection Kit (Invitrogen Life Technologies). The cells were transfected with the retroviral control vector pRetroX-Tight-Pur-luc (Clontech Laboratories Inc.) plasmid. RetroTight Pur vector without any genes was used as a plasmid control. The expression levels of the Luciferase gene induced by tetracycline in these cells were measured (using the Luciferase Assay System; Promega Corp., Madison, WI, USA), and the clone with the highest value was selected. Δp63β, previously cloned into the RetroTightPur vector, was transfected into selected stable T98G cells expressing rtTA most effectively, leading to the generation of T98G cells that overexpress Δp63β.

### 2.4. RNA isolation and reverse-transcription polymerase chain reaction (RT-PCR)

For RT-PCR validation of the mRNA, the total RNA was extracted using TRIzol Reagent (Invitrogen Life Technologies) from T98G and T98G cells that expressed Δp63β. The total RNA isolated was reverse-transcribed to cDNA using a RevertAid First Strand cDNA Synthesis Kit (Thermo Fisher Scientific Inc.), and validation was performed. PCR analysis was performed using a Taq DNA Polymerase Kit (Thermo Fisher Scientific Inc.) for the expression analyses. Three independent experiments were performed. The specific PCR primers are listed in the Table.

### 2.5. Western blot

Western blot was performed for expression analysis at the protein level. After the cells were passaged with 4 × 104 cells/well into 10 mL-cell culture plates, tetracycline was added to the cells every 24 h for 3 days. At the end of day 3, the proteins were extracted from the cells with protein lysis solution (50 mM, pH: 8 tris (Sigma-Aldrich Chemie GmbH, Steinheim, Germany), 5 mM of ethylenediaminetetraacetic acid (Merck KGaA, Darmstadt, Germany), 150 mM of sodium chloride (Merck KGaA), 0.5% octylphenoxypolyethoxyethanol (Thermo Fisher Scientific Inc.). The cells were lysed, followed by centrifugation to eliminate protein debris. Subsequently, the protein content was measured using a Bicinchoninic Acid Kit (Pierce Biotechnology Inc. Rockford, IL USA). Equal amounts of the proteins were run on a 10% polyacrylamide gel containing 1% sodium dodecyl-sulfate (SDS) (Bio-Rad, Hercules, CA, USA) and then transferred to the nitrocellulose membrane (Bio-Rad) by electro-transfer. Following transfer of the proteins, blocking was performed with a 5% milk blocking buffer. The membrane was maintained for 16 h by adding the antibody with the expression level that was to be investigated. After 16 h, the membrane was incubated with anti-antibody conjugated with horseradish peroxidase (HRP) in a shaker for 1.5 h, and protein bands were visualized using HRP (1/1000 antirabbit-antimouse; Advansta Inc., Menlo Park, CA, USA) substrate.

### 2.6. 3-(4,5-dimethylthiazol-2-yl)-2,5-diphenyltetrazolium bromide (MTT) assay

MTT assay, a spectrophotometric method, was used to determine the number of viable cells. After the cells were seeded at a density of 1 × 10^4^ cells/well in 96-well plates, tetracycline (100 ng/mL) was added to the cells every 24 h for 3 days. Following this, 10 μL of 5 mg/mL of MTT (MTT reagent; Sigma-Aldrich Chemie GmbH) solution was added to each well containing 100 μL of medium (final MTT concentration of 0.5 mg/mL). The plate was incubated at 37 °C with 5% CO_2_ for 2–4 h. After incubation, the solution was removed from each well, and 100 μL of dimethylsulfoxide (DMSO; Sigma-Aldrich Chemie GmbH) was added to each well to dissolve the formazan crystals. The absorbance was measured at 570 nm using a spectrophotometer.

### 2.7. Apoptosis test

A SensoLyte homogenous AFC Caspase-3/7 Assay Kit (AnaSpec Inc., Fremont, CA, USA), which is based on the determination of the activity of caspase-3 and caspase-7, which play an essential role in apoptosis, was used. After the cells were passaged with 4 × 10^4^ cells/well into 12-cell plates, tetracycline (100 ng/mL) was added to the cells every 24 h for 3 days. At the end of day 3, the cells were dissolved in the lysis solution and placed into microcentrifuge tubes. The cell suspension was shaken for 30 min at 4 °C. After centrifugation at 2.500x *g* for 10 min, the supernatant was placed on 96-cell plates and the caspase-3/7 substrate solution was added. After incubation for 30 min at room temperature, the fluorescence intensity was measured at Ex/Em = 380/500 nm.

### 2.8. β-galactosidase cell staining

A β-galactosidase Cell Staining Kit (Cell Signaling Technology Inc., Danvers, MA, USA) was used to detect the β-galactosidase activity, a known feature of senescent cells, at a pH of 6. After the cells were passaged with 4 × 10^4^ cells/well into 12-cell plates, tetracycline (100 ng/mL) was added to the cells every 24 h for 3 days. After washing the cells with phosphate-buffered saline (PBS), a fixation solution was added at the end of day 3. After 15 min, the cells were washed twice and incubated with β-galactosidase cell staining solution overnight at 37 °C. The cells were examined under a microscope for the development of blue coloring.

### 2.9. Cell cycle analysis by propidium iodide (PI) staining

In this method for cell cycle analysis using PI (Thermo Fisher Scientific Inc.), the fluorescent nucleic acid dye PI was used to determine the proportion of cells present in one of the three interphase stages. After the cells were passaged with 4 × 10^4^ cells/well into 10-mL cell culture plates, tetracycline (100 ng/mL) was added to the cells every 24 h for 3 days. At the end of day 3, the cells were collected, washed with PBS, and fixed in 70% ethanol for 30 min. After centrifugation, the cell pellet was washed with PBS, and RNase A (100 μg/mL) and PBS was added directly on top. PI (50 μg/mL) solutions were added and the cells were analyzed via flow cytometry.

### 2.10. Statistical analysis

The relative analysis of the gene expressions was performed using ImageJ 1.45s (US National Institutes of Health, Bethesda, MD, USA). Multiple t-test analysis was also performed using Graph Pad Instat (Graph Pad Inc., La Jolla, CA, USA).

## 3. Results

### 3.1. Obtaining T98G cell-expressing ΔNp63β

**Figure 1:**
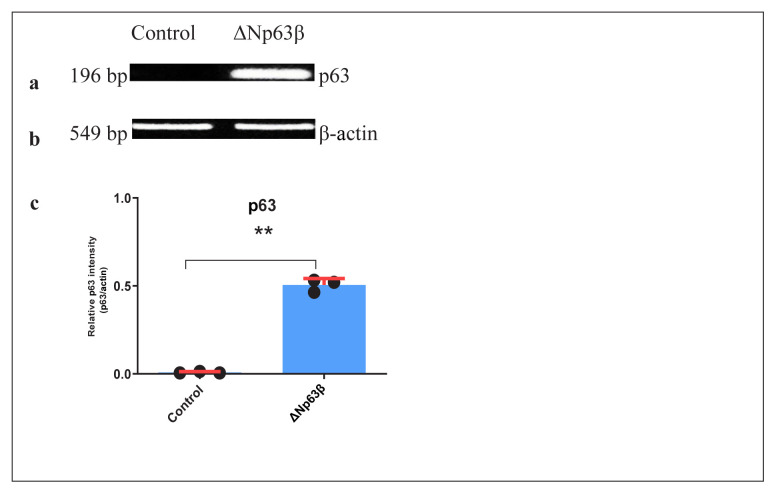
RT-PCR results of the p63 expression levels 72 h after induction with doxycycline of T98G glioma cells expressing ΔNp63β. a) Levels of p63 and b) β-actin were tested by PCR analysis. c) Graphic representation of the p63/β-actin level. Cells were extracted from ΔNp63β cells (control) that were not induced with doxycycline, and ΔNp63β cells that were induced with doxycycline for 72 h. β-actin was normalized. Data are presented as the mean ± standard error of the mean (SE) derived from a minimum of three independent experiments. ** p < 0.01

**Figure 2:**
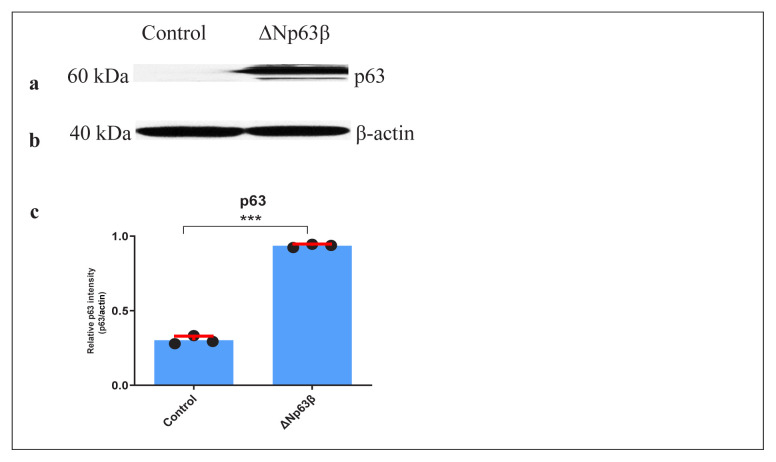
Western blot results of the p63 protein levels 72 h after induction with doxycycline of T98G glioma cells overexpressing ΔNp63β. a) The protein levels of p63 and b) β-actin were tested using western blot analysis. c) Graphic representation of the p63/β-actin level. Proteins were extracted from ΔNp63β cells that were not induced with doxycycline (control), and ΔNp63β cells that were induced with doxycycline for 72 h. β-actin was normalized. Data are presented as the mean ± SE derived from a minimum of 3 independent experiments. *** p < 0.001.

The pRetroX-Tet-On Vector System responding to tetracycline induction was used to continuously monitor the effect of the ΔNp63β on T98G cell functions. Initially, cell lines expressing rtTA were established using this vector system, followed by transfection of the pRetro-pur vector system containing ΔNp63β into this rtTA-expressing cell line. In this way, to examine the effects of overexpression of the ΔNp63β (Figures 1a–1c), the T98G cell line, with a promoter induced in the presence of tetracycline, was obtained (Figures 2a–2c).

### 3.2. Gene analysis to determine the genes responsible for the cell cycle control mechanism and proliferation

**Figure 3:**
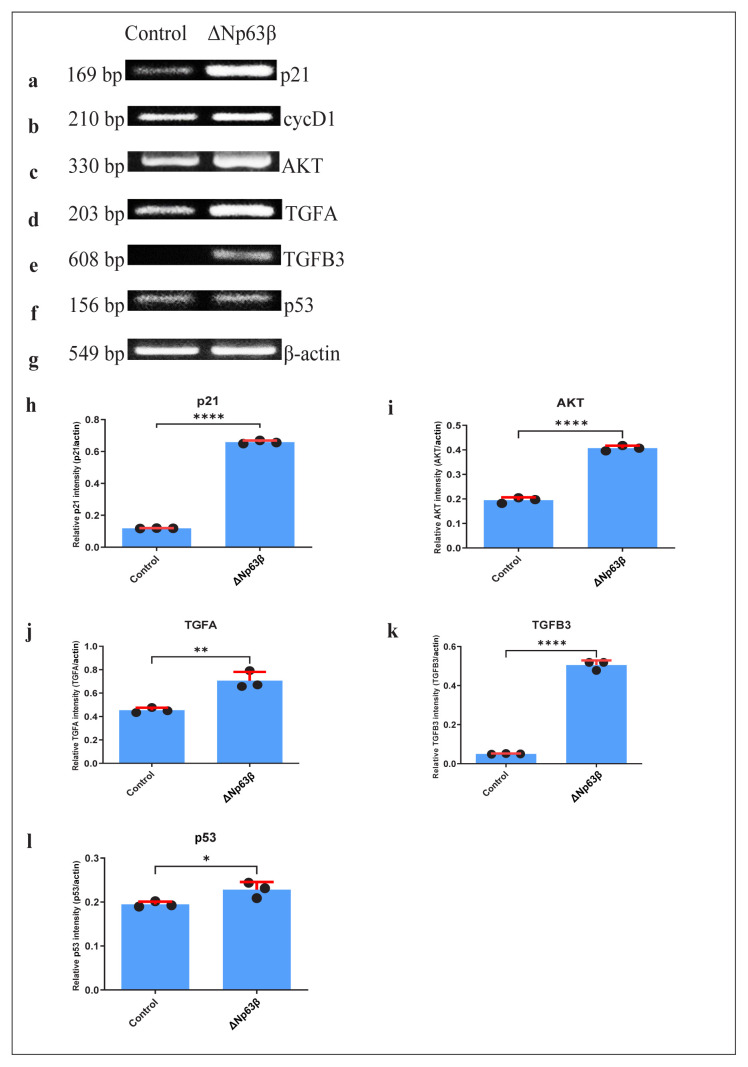
RT-PCR results of the cell cycle control mechanism and genes responsible for the proliferation expression levels 72 h after induction with doxycycline of T98G glioma cells overexpressing ΔNp63β. The cells were extracted from ΔNp63β cells that were not induced with doxycycline (control), and ΔNp63β cells that were induced with doxycycline for 72 h. In the cells overexpressing ΔNp63β, the p21, cycD1, AKT, TGFA, and TGFB3 expression increased, and there was no change in the p53 expression. a–g) PCR product in gel electrophoresis. h–l) Graph. β-actin was normalized. Data are presented as the mean ± SE derived from a minimum of three independent experiments. * p <0.05, ** p <0.01, **** p < 0.0001.

**Figure 4:**
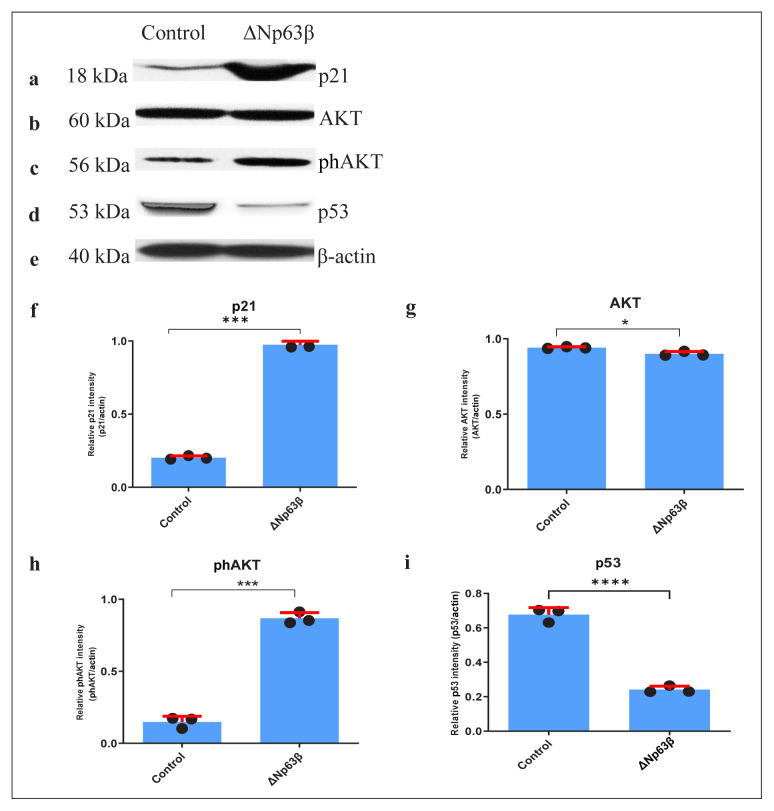
Western blot analysis results of the genes responsible for the cell cycle mechanism protein levels and proliferation protein levels 72 h after induction with doxycycline of T98G glioma cells overexpressing ΔNp63β. The cells were extracted from ΔNp63β cells that were not induced with doxycycline (control), and ΔNp63β cells that were induced with doxycycline for 72 h. In the cells overexpressing ΔNp63β, while the p21 and phAKT protein level increased, the p53 protein level decreased, and no change was observed in the AKT. a–e) Protein product in SDS-PAGE. f–i) Graph. β-actin was normalized. Data are presented as the mean ± SE derived from a minimum of 3 independent experiments. * p <0.05, *** p <0.001, **** p < 0.0001.

RNA extraction was performed from cells continuously expressing Δp63β in the presence of tetracycline, followed by PCR with cDNA, and western blot was performed with the proteins obtained from these cells. The expression levels of cyclin D1, p21, and p53, which are responsible for the cell cycle control mechanism, and AKT1, responsible for cell development, proliferation, and cell survival, including cancer, were examined. T98G cells that expressed Δp63β showed an increase of approximately 7-fold in p21 (Figures 3a and 3h) and an increase of approximately 40% in cyclin D1 (Figure 3b) when compared to those that did not express Δp63β. A 2-fold increase was detected in the AKT1 (Figures 3c and 3i) mRNA expression in T98G cells that expressed Δp63β. In T98G cells that express Δp63β, an increase of approximately 80% was detected in TGFα (Figures 3d and 3j), which is one of the epidermal growth factors, and an increase of approximately 10-fold was observed in TGFβ3 (Figures 3e and 3k). An increase of approximately 6-fold was detected in the p21 protein expression level in T98G cells expressing Δp63β (Figures 4a and 4f), consistent with the results obtained with the RT-PCR. Although there was no change in the AKT1 (Figures 4b and 4g) protein in T98G cells expressing Δp63β at the protein level, an increase of approximately 9-fold was detected in the expression of the phosphorylated AKT1 protein (Figures 4c and 4h). When the p53 expression was examined, although there was no change in the p53 mRNA level (Figures 3f and 3l), a decrease of more than 3-fold in the protein level was observed in T98G cells expressing Δp63β (Figures 4d and 4i).

**Figure 5:**
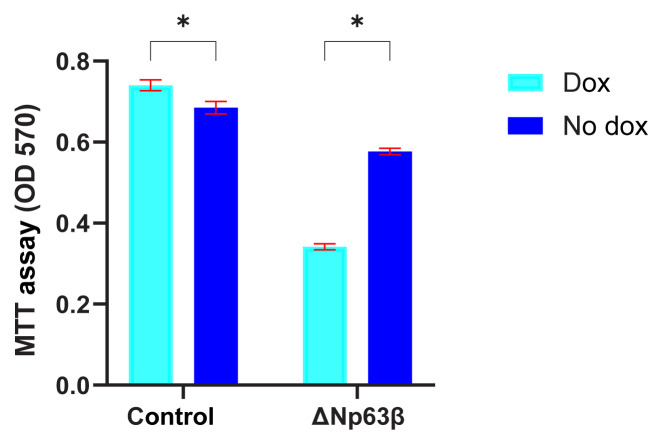
Representation of the ΔNp63β expression via MTT assay. Investigation of the effect of T98G glioma cells expressing ΔNp63β on cell viability by performing MTT test, repeated 3 times independently, at 72 h after induction with doxycycline. Cell viability in the ΔNp63β expressing cells decreased compared to control cells. Data are presented as the mean ± SE. * p < 0.05.

The MTT method, a spectrophotometric method used to determine the number of viable cells, was used to investigate the effects of the ΔNp63β isoform on cell viability. A decrease of approximately 50% was detected in T98G cells expressing ΔNp63β (Figure 5).

### 3.3. Expression analysis of apoptosis-related genes

**Figure 6:**
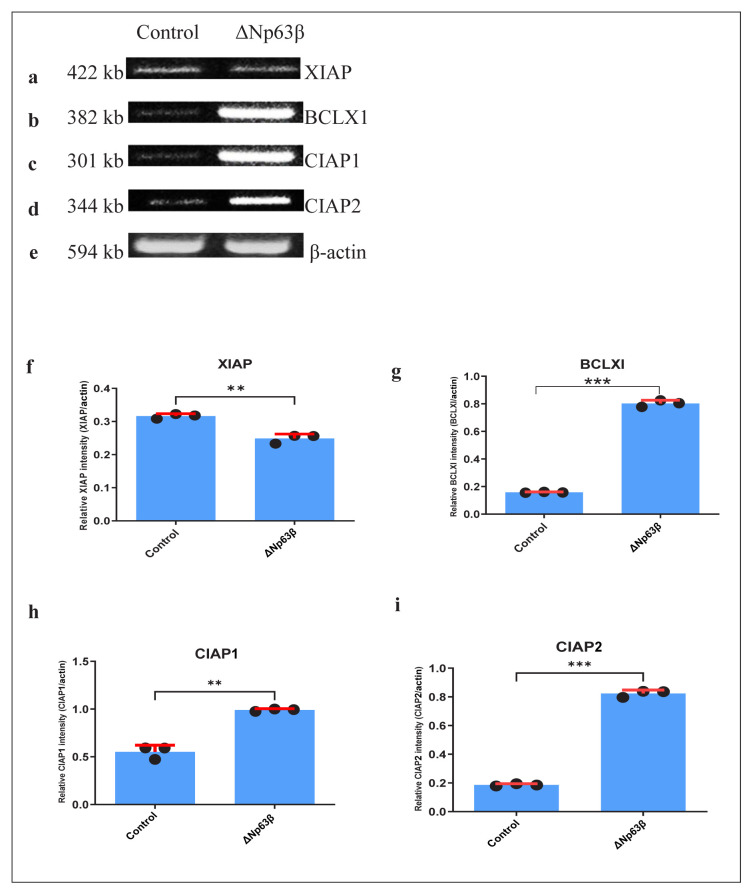
Representation of the apoptosis-related gene expression levels 72 h after induction with doxycycline of T98G glioma cells overexpressing ΔNp63β. The cells were extracted from ΔNp63β cells that were not induced with doxycycline (control), and ΔNp63β cells that were induced with doxycycline for 72 h. In cells overexpressing ΔNp63β, there was no change in the XIAP expressions, while the BCLX1, CIAP1, and CIAP2 expression levels increased. β-actin was normalized. a–e) PCR product in gel electrophoresis. f–i) Graph. Data are presented as the mean ± SE derived from a minimum of 3 independent experiments. ** p <0.01, *** p < 0.001.

Regarding apoptosis, the B-cell lymphoma-extra-large (BCLXL), Bcl-2-associated X protein (BAX), Bcl-2-associated death promoter (BAD), BH3-interacting domain death agonist (BID), caspase inhibitor apoptosis protein 1 (CIAP1), CIAP2, and X-linked inhibitor of apoptosis (XIAP) protein expression levels were examined. Although no changes were observed in the BAD, BAX, BID [Supplementary Figure (not statistically significant] and XIAP (Figures 6a and 6f) expression levels, there was an increase of approximately 5-fold in T98G cells expressing Δp63β in BCLX1 (Figures 6b and 6g), compared to those that did not express Δp63β. It is also noteworthy that there was an increase of 60% in CIAP1 (Figures 6c and 6h) and approximately 4-fold in CIAP2 (Figures 6d and 6i).

**Figure 7:**
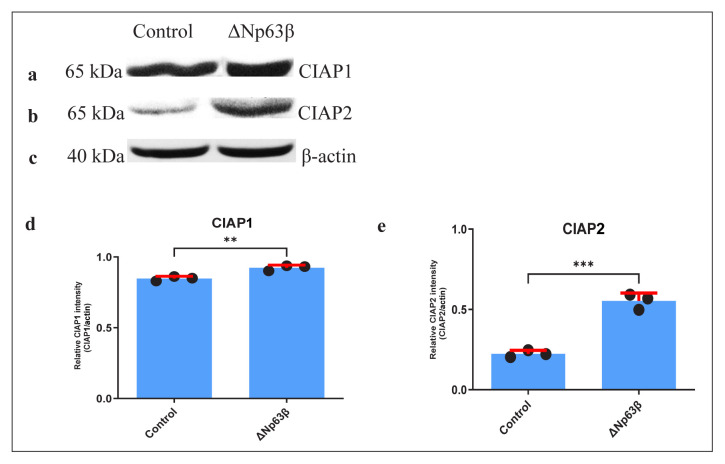
Representation of the apoptosis-related gene protein levels at 72 h after induction with doxycycline of T98G glioma cells overexpressing ΔNp63β. The cells were extracted from ΔNp63β cells that were not induced with doxycycline (control) and ΔNp63β cells that were induced with doxycycline for 72 h. The CIAP2 protein level was increased in the ΔNp63β-overexpressing cells, whereas no significant change was observed in the CIAP1 protein levels. β-actin was normalized. a–c) Protein product in SDS polyacrylamide gel electrophoresis (SDS-PAGE). d–e) Graph. Data are presented as the mean ± SE derived from a minimum of 3 independent experiments. ** p < 0.01, *** p < 0.001.

In T98G cells expressing Δp63β, an increase of approximately 20% was detected in CIAP1 (Figures 7a–7d) and an increase of approximately 40% in CIAP2 (Figures 7b and 7e) at the protein level.

**Figure 8:**
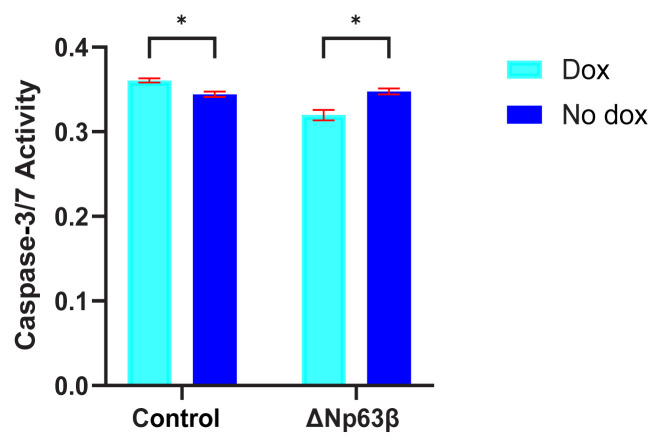
Representation of the ΔNp63β expression via apoptosis assay. Investigation of the effect of T98G glioma cells expressing ΔNp63β on apoptosis by performing apoptosis assay, repeated 3 times independently, at 72 h after induction with doxycycline. No significant change was observed. Data are presented as the mean ± SE. * p < 0.05.

The apoptosis effect of the ΔNp63β isoform on the cells was determined by looking at the activity of caspase-3 and caspase-7, which play essential roles in apoptosis. No significant changes could be detected in the T98G cells expressing ΔNp63β (Figure 8).

### 3.4. Effect of ΔNp63β isoform on cell cytotoxicity

**Figure 9:**
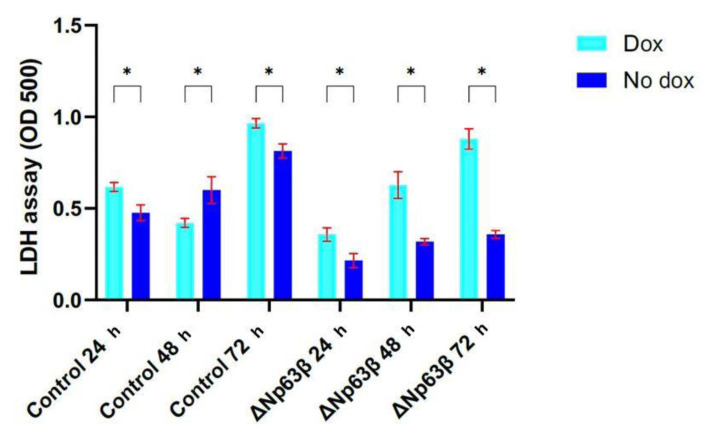
Representation of the ΔNp63β expression via LDH assay. Investigation of the cytotoxic effect of T98G glioma cells expressing ΔNp63β on the cells by performing the LDH test, repeated 3 times independently, at 72 h after induction with doxycycline. ΔNp63β expressing an increase in the LDH level was observed in the cells. Data are presented as the mean ± SE. * p < 0.05.

The LDH test was performed to show the direct cytotoxicity of the ΔNp63β isoform on the cells, and it was determined that death in T98G cells expressing ΔNp63β increased by approximately 50% (Figure 9).

### 3.5. Effect of ΔNp63β isoform on the cell cycle

**Figure 10:**
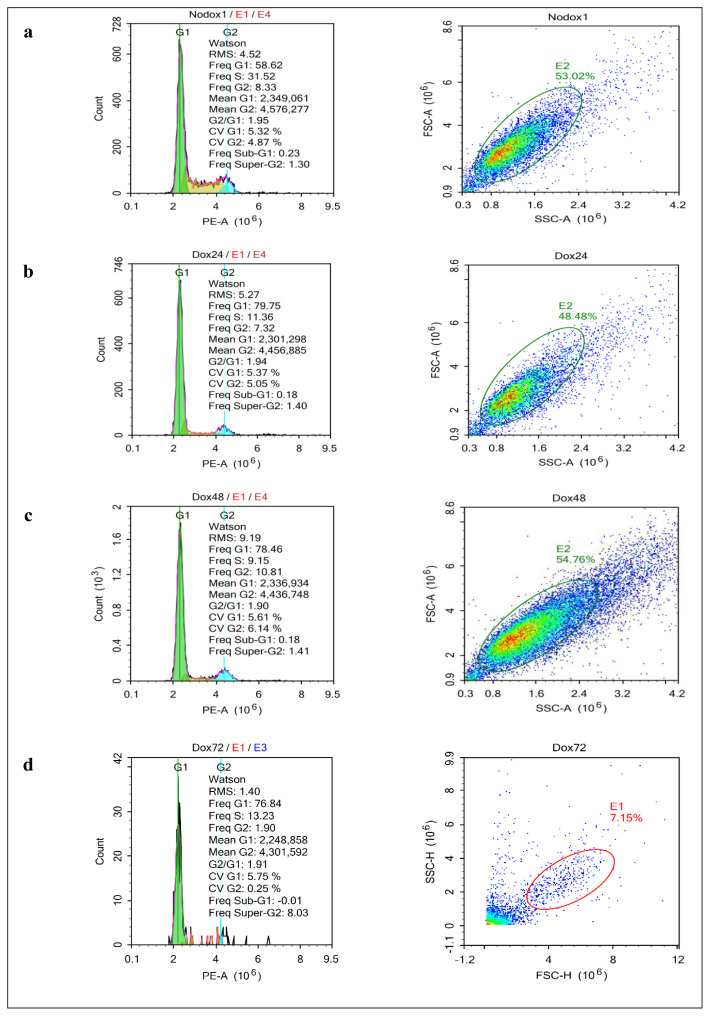
Cell cycle analysis of the T98G glioma cells with PI, in which staining was performed, and samples were analyzed by flow cytometry. a) Cell cycle of the uninduced cells (no dox), b) the cell cycle was induced to express ΔNp63β for 24 h (Dox24), c) 48 h (Dox48), and d) 72 h (Dox72). e) Graphs showing the distribution of the cell population according to cell phases. In cells expressing ΔNp63β, cell cycle arrest was observed in the G1 phase. Data are presented as the mean ± SE derived from a minimum of 3 independent experiments. * p < 0.05.

PI staining was performed to investigate the effect of the ΔNp63β isoform on the cell cycle. In T98G cells without Δp63β expression, the cell DNA content maintained the cell cycle phase distributions. In contrast, in T98G cells with Δp63β expression after 24 h induction with tetracycline, the balance of the cell DNA content changed. While an increase in the G1 phase and a decrease in S phase cells were observed, this ratio increased more in the presence of tetracycline for 48 and 72 h. The increase in the number of cells in the G1 phase with Δp63β expression in T89G cells indicates that Δp63β induces cell G1 phase arrest. In addition, the absence of any change in the number of sub-G1 cells suggests that Δp63β has no role in inducing apoptosis. (Figures 10a–10e).

### 3.6. Effect of ΔNp63β isoform on cell senescence

**Figure 11:**
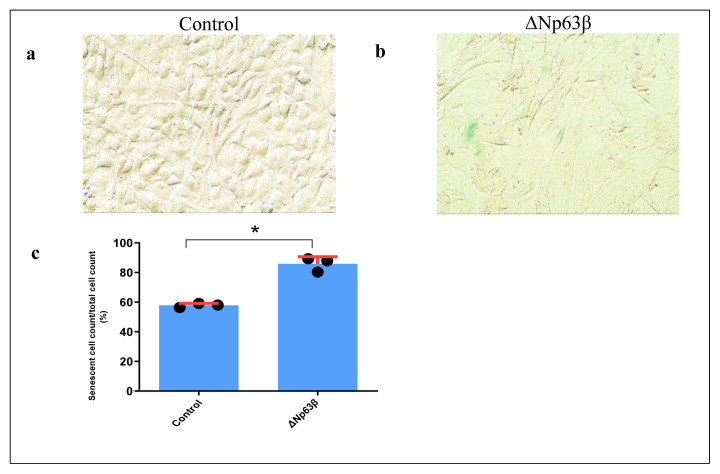
β-gal activity in the T98G glioma cells. a) Uninduced and b) dox-induced 72-h T98G glioma cells were subjected to in situ SA-β-gal staining at a pH of 6 and examined by bright field microscopy. In cells expressing ΔNp63β, staining due to cell senescence was observed to be increased. c) Graph showing the ratio of uninduced and dox-induced 72-h senescent cells to total cells. Data are presented as the mean ± SE derived from a minimum of three independent experiments. * p < 0.05.

The β-galactosidase cell staining method, which detects β-galactosidase activity, was used to investigate the effect of the ΔNp63β isoform on cell senescence. Contrary to the cells without ΔNp63β expression, senescence was observed in ΔNp63β cells expressed in the presence of tetracycline, and the growth of cells also stopped (Figures 11a–11c).

## 4. Discussion

Initially identified for its similarities to p53, p63 has since been recognized for its distinct characteristics across its multiple isoforms. These isoforms exhibit diverse and sometimes opposing functions, making the p63 gene a compelling subject of investigation. While much attention has been devoted to the α and γ isoforms, the β isoforms, including ΔNp63β, have received comparatively less scrutiny. The extensive involvement of p63 in fundamental cellular processes such as cell proliferation, DNA damage response, cell cycle regulation, apoptosis, ageing, and cancer metastasis highlights its critical importance for study. Literature specifically addressing the ΔNp63β isoform of p63, believed to be effective in T98G cells exhibiting GBM with epithelial differentiation, is notably lacking. With its innovative and novel approach, this study sought to uncover the unexplored roles of ΔNp63β in T98G cells, offering a fresh perspective on this intriguing gene that will stimulate further interest.

The tumor suppressor protein p53 is pivotal in regulating cell cycle and apoptosis. While the molecular pathways controlled by p53 family members are well-documented, ongoing studies persist in unravelling the physiological mechanisms underlying their actions. Notably, research indicates that the impact of p63 on cell cycle control mirrors that of p53 [3].

Crucial to mammalian cell cycle regulation, the cyclin-dependent kinase inhibitor p21 (p21WAF1/Cip1) stimulates cell cycle arrest. Its transcriptional activation is governed by p53 and operates specifically during the G1 phase of the cell cycle. Therefore, the ability of p21 to impede the cell cycle hinges on the activity of p53 [4,21]. In the current study, changes were observed in the p21 levels in cells expressing Δp63β, which further underscores the complex interplay between p63, p53, and cell cycle regulation.

Previous research showcased that TAp63 prompts an elevation in p21 levels independently of p53 [22]. Intriguingly, in the present study, cells expressing Δp63β exhibited a substantial increase in p21 despite a notable decrease in p53 protein levels in T989 cells expressing Δp63β. It was reported that TAp63 drives cell cycle arrest by transcriptionally elevating p21 and p57/Kip2 proteins, whereas ΔN isoforms suppress these promoters, thereby supporting continuous cell cycle progression [3,23].

Another study suggested that ΔNp63α can activate p21, but TA isoforms demonstrate a more substantial inducement of p21 [24]. However, contrary to this notion, the current study found a remarkable increase in p21 mRNA and protein levels in cells expressing Δp63β. Furthermore, in cells expressing Δp63β, cell death reached up to 50%, potentially causing cell cycle arrest at the G1 phase and incomplete cell replication, accompanied by a notable increase in p21 compared to nonexpressing cells. Similarly, cyclin D1, a key player in the G1 phase, exhibited a substantial increase in cells expressing Δp63β.

Furthermore, the present study revealed that cells expressing Δp63β exhibited a higher degree of senescence compared to those not expressing Δp63β, leading to a cessation in cell growth. These findings suggest a potential correlation between the activation of p21, regulated by tetracycline-induced Δp63β expression, and the inhibition of tumor development by inducing senescence.

An investigation into apoptosis inhibitor proteins was conducted in light of the implications for apoptosis associated with the Δp63β isoform. The crucial members of the inhibitor family of apoptosis proteins (IAPs) are cIAP1 and cIAP2, which act as cellular inhibitors of apoptosis by mitigating intracellular and extracellular death signals [25]. The experiments herein demonstrated a notable increase in mRNA and protein levels of cIAP1 and cIAP2 in cells expressing Δp63β. These findings strongly suggest that the Δp63β isoform possesses antiapoptotic characteristics. Moreover, results from the MTT and LDH tests corroborated the antiapoptotic properties of the Δp63β isoform. It is plausible that the observed cell death in cells expressing the Δp63β isoform stems from the cytotoxic effect exerted by this particular isoform.

Numerous studies have highlighted the divergent roles of p63 isoforms in cancer regulation. TAp63 has been recognized as a tumor suppressor, associated with cell death and cell cycle arrest, whereas ΔNp63 exhibits oncogenic characteristics [26], and its overexpression is noted in numerous cancer cases [10]. Despite the general acceptance of ΔNp63 as an oncoprotein in cancer regulation, the expression of TAp63 has been linked to a more favorable prognosis [27,28].

AKT emerges as a pivotal protein in signaling pathways involved in cell metabolism and cancer progression. It plays a crucial role in cell proliferation, viability, development, and angiogenesis, thereby promoting the continued proliferation and survival of cancer cells. Notably, both the mRNA levels and the phosphorylated form of AKT protein increased significantly in cells expressing Δp63β. Additionally, the elevated expression of genes associated with growth factors such as transforming growth factor beta 3 (TGF-β3), which plays roles in embryogenesis and cell differentiation, and the epidermal growth factor receptor TGFα, known to activate cell proliferation, development, and differentiation, alongside the upregulation of AKT1 and phAKT, collectively support the potential oncogenic nature of the Δp63β isoform. In conclusion, the present study uncovered an unexpected observation that challenges the prevailing literature. The overexpression of ΔNp63β in T98G cell lines surprisingly resulted in substantial cell death. The findings of this study suggest that the impact of ΔNp63β on cell death in T98G cells may not rely on apoptosis but instead involve necrosis-associated mechanisms. This surprising twist challenges our understanding of p63 isoforms and their roles in cancer. This unexpected discovery opens up new avenues for research and potential therapeutic strategies in cancer treatment.

## Supplementary results

Supplementary Figure Representation of the apoptosis-related genes expression levels and protein levels 72 h after induction with doxycycline of T98G glioma cells overexpressing ΔNp63β. Cells were extracted from ΔNp63β cells that were not induced with doxycycline (control) and ΔNp63β cells that were induced with doxycycline for 72 h. The CIAP2 protein level increased in the ΔNp63β-overexpressing cells. Data are presented as the mean ± SE derived from a minimum of 3 independent experiments (not statistically significant).

## Figures and Tables

**Table:** Sequence information for specific primers.

Primer	Sequence (5′→ 3′)	Product size	Purpose of use
4-5-6 F KPN1 R	TACAGCTAACATGTTGTACCT TGTCGTGAATTCAGTGCCAAC	631 bp	Partial cloning
KPN1 F MFE1 R	GTGTGCTGGTACCTTATGAG CTGTTGCTGTTGCCTGTA	467 bp	Partial cloning
MFE1 F 2–5 R	CAGTACCTTCCTCAGCACAC CCTTCCAGATCGCATGTCG	427 bp	Partial cloning
4-5-6 F 2–5 R	TACAGCTAACATGTTGTACCT CCTTCCAGATCGCATGTCG	1427 bp	Partial cloning
β-actin F β-actin R	AGAAAATCTGGCACCACACC AGGAAGGAAGGCTGGAAGAG	594 bp	Expression analysis
p63all F p63all R1	CTCCACCTTCGATGCTCTCT AGATCAAGGTGATGACCCCAC	196 bp	Expression analysis
BAD F BAD R	TCCAGATCCCAGAGTTTGAG TGTGGCGACTCCGGATCTCCACAG	216 bp	Expression analysis
BAX F BAX R	TTGCTTCAGGGTTTCATCCAG TGCAGCTCCATGTTACTGTC	155 bp	Expression analysis
BID F BID R	TACCCTAGAGACATGGAGAAG TGGCTAAGCTCCTCACGTAG	160 bp	Expression analysis
P21 F P21 R	AAGACCATGTGGACCTGTCA GGCTTCCTCTTGGAGAAGAT	169 bp	Expression analysis
AKT1 F AKT1 R	ATGAGCGACGTGGCTATTGTGAAT GAGGCCGTCAGCCACAGTCTGGATG	330 bp	Expression analysis
TGFA F TGFA R	ACACTCAGTTCTGCTTCCATG ACATGTGATGATAAGGACAGC	203 bp	Expression analysis
XIAP F XIAP R	TCAGCATCAACACTGGCACGAG TCTCTTGGGGTTAGGTGAGCATAG	422 bp	Expression analysis
CIAPI F CIAPI R	GCACATTCATTATCTCCCACCTTG CATCATCCTTTGGTTCCCAGTTAC	301 bp	Expression analysis
CIAP2 F CIAP2 R	CCTCTCAGCCTACTTTTCCTTCTTC CATAGCATTATCCTTCGGTTCCC	344 bp	Expression analysis
BCLX1 F BCLX1 R	ACTGAATCGGAGATGGAGACCC TGAAGAGTGAGCCCAGCAGAAC	382 bp	Expression analysis
CYCD1 F CYCD1 R	ATCTACACCGACAACTCCATC GAACTTCACATCTGTGGCAC	210 bp	Expression analysis
P53 F P53 R	CTTGCCACAGGTCTCCCCAA AGGGGTCAGAGGCAAGCAGA	156 bp	Expression analysis
M13 F M13 R	GTTTTCCCAGTCACGAC TATGACCATGATTACGCCAAG	-	Sequence analysis
