# Supplementary figures and images for: Effect of ΔNp63β on cell cycle and apoptosis in T98G cells
Source: Turk J Med Sci. 2024 Jun 20;54(6):1355–68. doi: 10.55730/1300-0144.5919 (PMC11673646; doi:10.55730/1300-0144.5919)

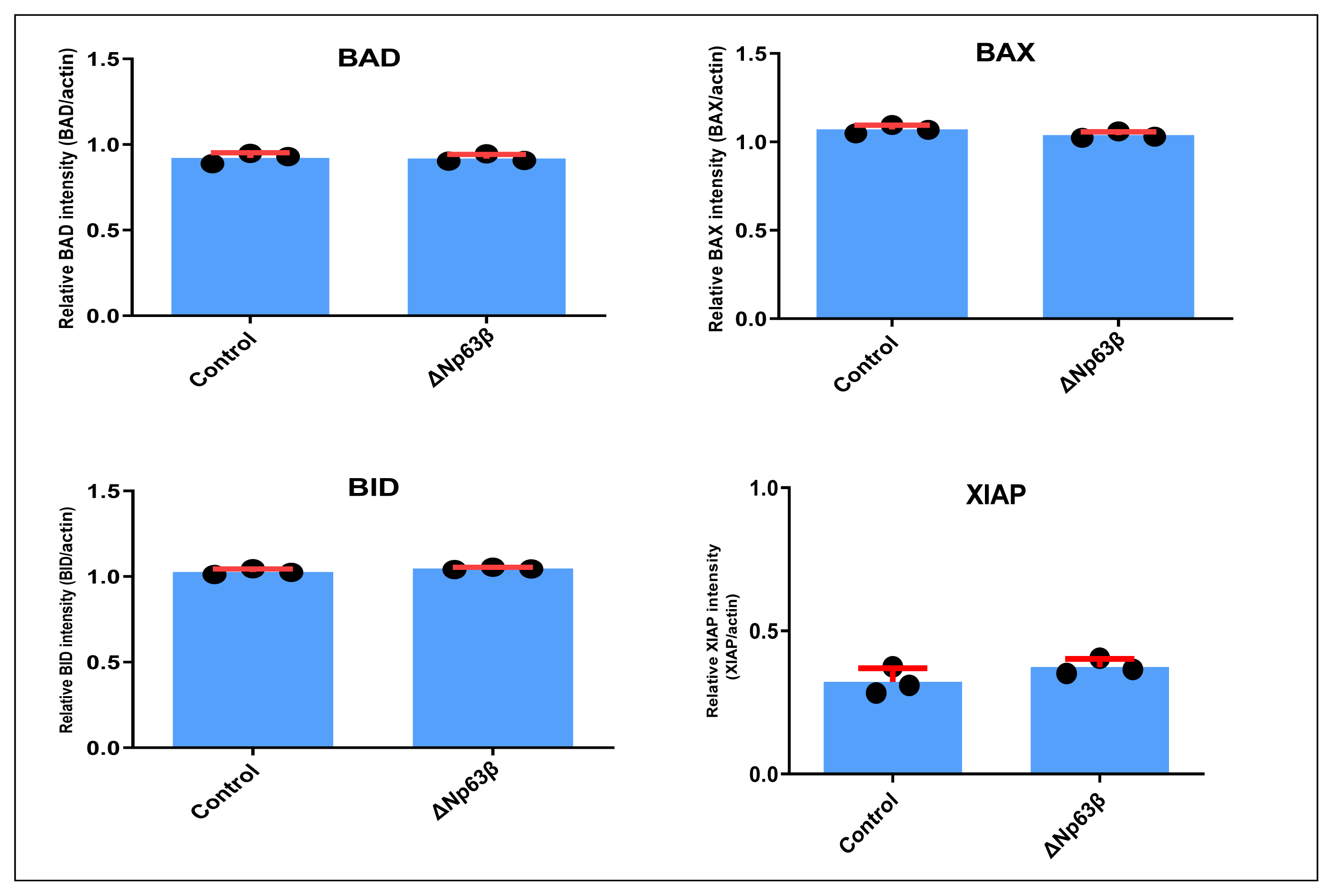

Supplement: Supplementary Figure — Representation of the apoptosis-related genes expression levels and protein levels 72 h after induction with doxycycline of T98G glioma cells overexpressing ΔNp63β. Cells were extracted from ΔNp63β cells that were not induced with doxycycline (control) and ΔNp63β cells that were induced with doxycycline for 72 h. The CIAP2 protein level increased in the ΔNp63β-overexpressing cells. Data are presented as the mean ± SE derived from a minimum of 3 independent experiments (not statistically significant). [file tjmed-54-06-1355s1.tif]
